# Perioperative mortality of head and neck cancers

**DOI:** 10.1186/s12885-021-07998-z

**Published:** 2021-03-09

**Authors:** Yannan Wang, Mengxue Wang, Yan Tang, Bincan Sun, Kai Wang, Feiya Zhu

**Affiliations:** 1 Department of Oral and Maxillofacial Surgery, The Second Xiangya Hospital of Central South University, Changsha, Hunan China; 2 Department of Nursing, The First Hospital of Hunan University of Chinese Medicine, Changsha, Hunan China

**Keywords:** Head and neck cancers, Perioperative mortality, Prior radiotherapy, Previous chemotherapy, Infection, Hypertension, Diabetes

## Abstract

**Background:**

Head and neck cancers are aggressive cancers, most clinical studies focused on the prognosis of patients with head and neck cancer. However, perioperative mortality was rarely mentioned.

**Methods:**

A retrospective analysis was performed using all head and neck cancer patients admitting in the Department of Oral and Maxillofacial Surgery of the Second Xiangya Hospital, Central South University from January 2010 to December 2019. The analysis of overall survival and progression-free survival were performed using the Kaplan–Meier method, and cross tabulation with chi-squared testing was applied to analyze the difference in parameters between groups.

**Results:**

From January 2010 to December 2019, a total of 6576 patients with head and neck cancers were admitted to our department and 7 died in the hospital, all of whom were middle-aged and elderly patients including 6 males and 1 female. The perioperative mortality rate (POMR) was about 1‰. The causes of death included acute heart failure, rupture of large blood vessels in the neck, hypoxic ischemic encephalopathy due to asphyxia, respiratory failure and cardiopulmonary arrest.

**Conclusion:**

Preoperative radiotherapy, previous chemotherapy, hypertension, diabetes, advanced clinical stage and postoperative infection are risk factors for perioperative mortality of head and neck cancer.

## Background

Head and neck cancers (HNC) are aggressive cancers that usually have a poor prognosis. There are more than 500,000 new cases of HNC worldwide every year [1, 2], with 40,000 new cases and 7890 deaths reported in the United States [3]. Despite the therapeutic advances in the last 30 years, the 5-year survival rate of patients with HNC has not obviously improved [4, 5]. Surgical resection remains the primary treatment approach for HNC, including cases of recurrence [6]. With the improvement of treatment modalities and anesthesia technology in recent years, the perioperative complications in HNC patients have decreased significantly. However, perioperative mortality (POM) still occurs. Most studies of HNC reported a perioperative mortality rate (POMR) ranging from 1 to 3 deaths per 200 patients [7–9]. The perioperative death of HNC patients is always caused by myocardial infarction, hematoma, pulmonary embolism, lung infection, and/or any other factors [10, 11]. Previous clinical studies have focused on the prognosis of HNC patients with limited attention on perioperative deaths [12]. Studying the clinical manifestations and perioperative death of HNC patients will help doctors realize the risk of POM and take timely measures to reduce its occurrence. Moreover, a better understanding of POM will help doctors choose the best personalized treatment when facing patients with advanced cancers or poor surgical tolerance: choose the more radical surgical treatment that may lead to perioperative death or the palliative treatment with less risk but having poor response.

In this study, we systematically reviewed the medical records of 6576 HNC patients who received or planned to undergo surgery in our hospital from January 2010 to December 2019. Among them, 7 patients died during perioperative period and POMR was about 1‰. Infection and hypertension were correlated with poor overall survival of HNC patients. Preoperative radiotherapy, previous chemotherapy, hypertension, diabetes, advanced clinical stage and postoperative infection are risk factors for perioperative death of HNC.

## Materials and methods

### Study design and samples

In this study, we designed and implemented a retrospective study which was approved by the Ethics Committee of Second Xiangya Hospital of Central South University. This study includes all patients admitted to the Department of Oral and Maxillofacial Surgery of the Second Xiangya Hospital, Central South University from January 2010 to December 2019. Inclusion criteria of patients includes: 1. Intend to or have undergone general anesthesia surgery (tumor resection). 2. Pathologically diagnosed as head and neck cancer. For further evaluation of risk factors for perioperative death, enrolled patients should meet the following criteria: 1. With or without a definite diagnosis of diabetes or hypertension. 2. With or without clear diagnosis of underlying diseases including diabetes or hypertension, whether they have received radiotherapy and/or chemotherapy before surgery. 3. Have complete records of treatment process and postoperative follow-up. The patients who received other surgical treatments were excluded.

### Data collection methods

One doctor collected and sorted the patients’ data from the hospital’s electronic database which contains prior medical records and postoperative follow-up information. The data was double-checked by the other doctor.

### Demographics and clinical data

The data collated in the present study included demographic distribution, pathology, clinical imaging and treatment. For demographics and comorbidities, age, gender, status of smoking and alcohol drinking, hypertension, diabetes, clinical stage and ASA class were considered. Preoperative parameters included primary lesion, history of radiotherapy and/or chemotherapy, tumor recurrence and HNC operation history. Parameters during surgery consisted of airway management, neck dissection, skin flap types, operation time, blood loss and transfusion. The postoperative parameter of stage classification was ultimately diagnosed based on the pathologic examination of the primary tumor and lymph nodes intraoperatively removed during the operation.

### Statistical analysis

All analyses were performed with the SPSS 25.0 software (SPSS Inc., USA), R, version 3.6.1 (R Foundation for Statistical Computing, Vienna, Austria), and GraphPad Prism software version 7.0 (GraphPad Software, Inc., La Jolla, CA, USA). The analysis of overall survival and progression-free survival were calculated using the Kaplan–Meier method. Cross tabulation with chi-squared testing was applied to analyze the difference in parameters between groups in Table 3. *P*-value< 0.05 was considered statistically significant.

## Results

### The POMR of HNC patients was about 1‰

From January 2010 to December 2019, a total of 6576 HNC (4015were male and 2561 were female) patients admitted to our hospital who were ready to accept or had undergone surgery, with an average age of (48.56 ± 3.21) years. The other demographics and comorbidities details of the 6576 OSCC patients see Table 1. Seven patients (6 males and 1 female) died during perioperative period and the POMR was about 1‰, aged from 46 to 76 years, with an average age of (60.71 ± 8.12) years. Among them, 5 cases had comorbidities, including 3 cases of hypertension, 1 case of diabetes, 1 case of hypertension, diabetes, hypertensive heart disease and Hypercholesterolemia. In addition, 1 case had previous head and neck tumor-related surgery, and 3 case had received radiotherapy before surgery. For the 7 deaths, 3 patients died of cervical artery rupture and hemorrhage. One patient died of acute heart failure, cardiac arrest, severe pneumonia, and ischemic hypoxic encephalopathy. The demographics and comorbidities details of the 7 deaths see Table 2.

**Table 1:** Clinical and demographic characteristics of the patients (*n* = 6576)

Variable		Patients numbers (n)
Gender	Male	4015
	Female	2561
Age	>60	2703
	≤60	3873
Hypertension	Yes	1124
	No	4498
	NA	954
Diabetes	Yes	483
	No	4945
	NA	1148
Smoking	Yes	3504
	No	2033
	NA	1039
Drinking	Yes	2966
	No	2713
	NA	897
Stage	I-II	2457
	III-IV	2870
	NA	1249
ASA Class	I-II	2346
	III-IV	3773
	NA	457
Preoperative radiotherapy	Yes	462
	No	5482
	NA	632
Previous chemotherapy	Yes	165
	No	5567
	NA	844
Tumor recurrence	Yes	475
	No	5601
	NA	500
Neck dissection	Unilateral	557
	Bilateral	4648
	No	1371
Airway management	Yes	4487
	No	1389
	NA	700
Operation time (min)	>600	1543
	≤600	4681
	NA	352
Postoperative infection	Yes	445
	No	3267
	NA	2864
Operation blood loss (ml)	>600	3011
	≤600	2652
	NA	913
Free flap repair	Yes	4192
	No	1878
	NA	506

**Table 2:** Demographics, comorbidities and preoperative parameters of the 7 perioperative deaths

Patient ID	Sex	Age	Smoking	Drinking	Tumor recurrence	Previous operation	Previous radiotherapy	Previous chemotherapy	Comorbidities
1	Male	68	Yes	Yes	No	No	No	No	No
2	Male	63	Yes	Yes	No	No	Yes	Yes	Hypertension
3	Male	51	Yes	Yes	No	No	Yes	No	Hypertension
4	Female	76	No	No	No	No	No	No	No
5	Male	61	No	Yes	No	No	No	No	Diabetes
6	Male	46	Yes	Yes	No	No	No	No	Hypertension, Diabetes, hypertensive heart disease, hypercholesterolemia
7	Male	60	Yes	Yes	Yes	Yes	Yes	No	Hypertension

### Case presentation

#### Patient 1

Male, 68 years old, admitted to hospital on March 18, 2013, pathologically diagnosed as “Left soft palate squamous cell carcinoma”. Four days after admission, “Extended tumor resection combined with cervical lymph node clearance + left anterolateral femoral myocutaneous flap transfer repair + tracheotomy” was performed under general anesthesia. On the 4th day after the operation, the patient developed active bleeding in the mouth and nose, and dark red liquid could be drawn from the gastric tube. The patient died after 2 h’ rescue. The causes of death were: 1. Respiratory obstruction. 2. Hemorrhagic shock.

#### Patient 2

Male, 63 years old, admitted to hospital on December 9, 2013, diagnosed as “Left soft palate squamous cell carcinoma”, had a history of hypertension for more than 10 years. Four days after admission, “Extended tumor resection + left anterolateral femoral myocutaneous flap transfer repair + tracheotomy” was performed under general anesthesia. On the 2nd day after the operation, the patient developed “loss of consciousness, cyanosis of the lips, no spontaneous breathing, no heartbeat and disappearance of carotid artery beats”, and died 1.5 h’ after rescue. Cause of death: Cardiac respiratory arrest.

#### Patient 3

Male, 51 years old, admitted to hospital on September 16, 2014, diagnosed as “Left Tonsil Squamous Cell Carcinoma”, had a history of hypertension for more than 13 years, and had received radiotherapy and chemotherapy before the admission. Nine days after admission, “Extended tumor resection combined with cervical lymph node clearance + left anterolateral thigh muscle skin flap transfer repair + tracheotomy” was performed under general anesthesia. A flap crisis occurred on the 4th day after surgery, and the “pectoralis major myocutaneous flap transplantation” was performed again. On the 3rd day after the second operation, pus exuded from the right neck wound, which gradually increased. Bacterial culture of wound secretions showed: Enterobacter aeruginosa, *Pseudomonas aeruginosa* infection. On the 12th day after the second operation, the patient had massive bleeding from the neck wound, mouth and nose, and died 1.5 h later. The cause of death: bleeding from large blood vessels in the neck leads to respiratory and circulatory failure.

#### Patient 4

Female, 76 years old, admitted to the hospital on October 6, 2015, diagnosed as “Left Tongue Cell Carcinoma”. Magnetic resonance imaging (MRI) showed the tumor invaded the internal carotid artery. Considering that the internal carotid artery may need to be ligated during the operation, a “ballon occlusion test (BOT)” was performed before the operation. Nine days after admission, “Extended tumor resection combined with left anterolateral thigh musculocutaneous flap transfer repair+ tracheotomy” was performed under general anesthesia. Five days after the operation, the patient had bleeding from the neck wound. Emergency surgery indicated internal carotid artery rupture, and the rupture was located where the balloon was placed. After the comprehensive consideration of the advantages and disadvantages of the two treatment options of vascular anastomosis and internal carotid artery ligation, internal carotid artery anastomosis was selected after consultation with the patient’ families. On the 2nd day after the emergency operation, the patient developed neck bleeding again and died after half an hour. The cause of death: hemorrhage of large blood vessels in the neck.

#### Patient 5

Male, 61 years old, admitted to hospital on February 5, 2017, diagnosed as “Left oropharyngeal squamous cell carcinoma”, had a history of diabetes for more than 9 years. Four days after admission, “Extended tumor resection combined with left anterolateral thigh musculocutaneous flap transfer repair+ tracheotomy” was performed under general anesthesia. Pulmonary infection occurred on the 11th day after surgery. On the 12th day after surgery, the patient developed symptoms of loss of consciousness and shortness of breath, heart rate increased to 178 beats/min, blood oxygen saturation was 74%, and blood pressure was 115/67 mmHg. The patient died 1 h despite all treatments. The cause of death: heart failure.

#### Patient 6

Male, 46 years old, admitted to the hospital on March 1, 2018, diagnosed as “Left tongue squamous cell carcinoma”. Eight days after admission, “Extended tumor resection combined with left anterolateral thigh musculocutaneous flap transfer repair” was performed under general anesthesia. Three days after the operation, the patient developed neck wound infection. On the 6th day after the operation, the patient developed irritability, poor breathing, dark red blood flowing out of the mouth, obvious swelling of the neck wound, and bleeding from the incision. After opening the neck wound at the bedside, “dark red blood clots and active bleeding” were found. An emergency tracheotomy was performed immediately. During the tracheotomy, a cardiac arrest occurred and chest compressions were performed. The heartbeat recovered 36 min later. The patient underwent “debridement and hemostasis” of neck wound under general anesthesia, and was sent to ICU for further sub-hibernation treatment after the operation. The patient developed brain death 12 days later. The cause of death: ischemic hypoxic encephalopathy.

#### Patients 7

Male, 60 years old, admitted to the hospital on December 24, 2018, diagnosed as “Postoperative recurrence of left oropharyngeal squamous cell carcinoma”. The patient had a history of 13 years of hypertension and received 3 months of radiotherapy before admission. The physical examination indicated that the patient had cachexia, electrolyte imbalance, and hypoproteinemia. Two days after admission, the patient developed dyspnea with a blood oxygen saturation of 85% and was transferred to ICU for further treatment. Four days after admission, the patient developed lung infection (severe pneumonia) and type I respiratory failure. Twelve days after admission, the patient’s families gave up treatment, and the patient died 2 h after removing the ventilator. The causes of death: severe pneumonia and type I respiratory failure.

The summary of intraoperative and postoperative parameters of the 7 perioperative deaths could been seen in Table 3.

**Table 3:** Intraoperative and postoperative parameters of the 7 perioperative deaths

Patient ID	Stage	Tumor site	ASA class	Operation time (min)	Neck dissection	Airway management	Local wound infection	Etiology of perioperative mortality
1	III	Left soft palate	III	850	Unilateral	Tracheotomy	Yes	Cervical artery rupture and bleeding, airway obstruction
2	III	Left soft palate	III	350	Unilateral	Tracheotomy	No	Cardiac arrest
3	IV	Right tonsil	III	760	Bilateral	Tracheotomy	Yes	Cervical artery rupture and bleeding
4	IV	Left tongue	III	585	Bilateral	Tracheotomy	Yes	Cervical artery rupture and bleeding
5	III	Left mouth pharynx	II	580	Unilateral	Tracheotomy	No	Acute heart failure, lung infection
6	III	Left tongue	II	410	Unilateral	No	Yes	Ischemic hypoxic encephalopathy (brain death), lung infection
7	IV	Left neck lymph node	III	No	No	No	No	Severe pneumonia, type I respiratory failure

In addition, all patients were routinely given antibiotics one before surgery and 5 days after surgery to prevent infection in our department. For patients with diabetes, perioperative blood glucose was controlled in 6.7 ~ 10.0 mmol/L which was consistent with most scholars’ recommendations [13]. As for patients with hypertension, the systolic pressure 140 mmHg or less and diastolic pressure 90 or less are better blood pressure controls.

### Preoperative radiotherapy, previous chemotherapy, hypertension, diabetes, advanced clinical stages and postoperative infection are risk factors for perioperative death of HNC

In order to further evaluate the risk factors of perioperative death, we screened out 1795 patients with complete data from 6576 patients (Table 4). The results of cross tabulation with chi-squared testing showed that previous chemotherapy, prior radiotherapy, postoperative infection, diabetes, advanced clinical stages and hypertension are risk factors for perioperative death. And active airway management (including postoperative tracheal intubation and tracheotomy) can reduce the risk of perioperative death (Table 4).

**Table 4:** Clinical and demographic characteristics of the patients (*n* = 1795)

Variable		Dead (*n* = 7)	Alive (*n* = 1688)	χ ²	*P*
Gender	Male	6	1082	1.419	0.236
	Female	1	606		
Age	>60	5	694	2.658	0.103
	≤60	2	994		
Hypertension	Yes	4	417	3.956	0.042*
	No	3	1271		
Diabetes	Yes	2	136	3.978	0.046*
	No	5	1552		
Smoking	Yes	5	1105	0.109	0.742
	No	2	583		
Drinking	Yes	6	975	2.24	0.13
	No	1	713		
Stage	I-II	0	825	6.681	0.01*
	III-IV	7	863		
ASA Class	I-II	2	820	1.123	0.289
	III-IV	5	868		
Preoperative radiotherapy	Yes	3	112	5.382	0.02*
	No	4	1576		
Previous chemotherapy	Yes	1	68	10.861	0.001*
	No	6	1620		
Tumor recurrence	Yes	1	156	3.106	0.078
	No	6	1532		
Neck dissection	Unilateral	4	153	3.441	0.179
	Bilateral	2	1348		
	No	1	187		
Airway management	Yes	5	1584	5.972	0.014*
	No	2	104		
Operation time (min)	>600	2	617	0.192	0.661
	≤600	5	1071		
Postoperative infection	Yes	4	306	7.147	0.007*
	No	3	1382		
Operation blood loss (ml)	>600	5	1025	0.335	0.562
	≤600	2	663		
Free flap repair	Yes	6	1304	0.16	0.68
	No	1	334		

### Hypertension, postoperative infection and clinical stage are significantly related to poor OS in HNC patients

**Fig. 1:**
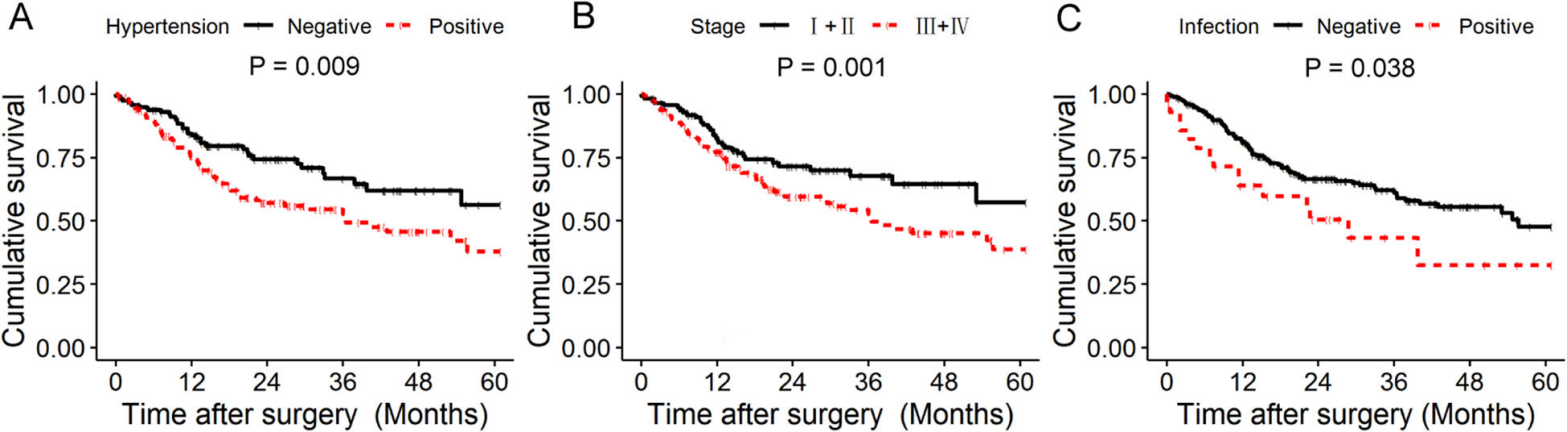
Overall survival of 1295 patients with OSCC who: **a** with or without hypertension. **b** clinical stage and **c** with or without postoperative wound infection. Estimated by Kaplan–Meier method. *P* value < 0.05 was considered statistically significant

To further evaluate the relationship between the prognosis of HNC and preoperative radiotherapy, previous chemotherapy, hypertension, diabetes, clinical stage and postoperative infection, we selected patients from 1295 patients who underwent surgery from January 2010 to December 2014 and evaluated their five-year survival rate. It was found that hypertension, postoperative infection and clinical stage were significantly related to poor OS (Fig. 1a, b and c).

## Discussion

In recent years, with the continuous advancement of diagnosis and treatment, the perioperative mortality of HNC patients has gradually decreased, from 6 to 8% in the 1970s [14, 15] to 0.5–1.5% in the 2000s [7–9]. Although the mortality rate has been declining, as the incidence of HNC has increased, its absolute number should not be underestimated especially in developing countries [16, 17]. In the present study, we found from January 2010 to December 2019, the POMR of HNC patients in our hospital was about 1‰ which was significantly lower than 0.5% ~ 1.5% mortality rate reported in 2007 [8]. Moreover, we found previous chemotherapy, prior radiotherapy, postoperative infection, diabetes, advanced clinical stages and hypertension are risk factors for perioperative death. And active airway management (including postoperative tracheal intubation and tracheotomy) can reduce the risk of perioperative death.

Previous studies indicated that: chemotherapy [18], radiotherapy [14] and postoperative wound infections [19, 20] may cause carotid artery damage. In the late stage of radiotherapy for head and neck malignant tumors, radiation and chemotherapy drugs could cause inflammatory reactions of carotid artery endothelial cells, vascular intima thickening, media necrosis and adventitia fibrosis [21–23]. As a result, carotid artery wall becomes stiff and less elastic. And patients with radiotherapy and/or chemotherapy have an increased risk of infection and difficulty in wound healing during hospitalization. Here, we found that rupture of carotid artery was the major cause of perioperative death. The direct cause of 3 deaths (patient1, patient 3, patient 4) was rupture of carotid artery. For example, patient 3 had radiotherapy before the operation, the radiotherapy dose reached 65Gy. The free flap necrosis occurred on the 4th day after the first operation, and the “pectoralis major myocutaneous flap transplantation” was performed again. On the 3rd day after the second operation, wound infection occurred in the right neck. On the 12th day, the patient died due to the massive hemorrhage in the neck wound, mouth and nose. After the retrospective analysis of the 3 deaths, we thought preoperative radiotherapy and skin flap necrosis increased the risk of neck infection which leads to invasive carotid arterial wall inflammation. And tumor invasion also causes damage to the arterial wall, eventually the patient died due to the rupture of carotid artery. It should be noted that preoperative radiotherapy is also a risk factor for necrosis of free flaps.

There is no literature report on the relationship between hypertension and HNC perioperative mortality. In this study, we conclude that hypertension is one of the risk factors for HNC perioperative mortality. After reviewing the medical records of the 7 patients, we found that a total of 3 patients (patient 2, patient 3, patient 7) had hypertension. The patient 2 had a history of hypertension, and the preoperative blood pressure was controlled at around 135/80 mmHg. The preoperative cardiac color Doppler ultrasound showed changes in the heart caused by hypertension, decreased aortic elasticity and normal systolic function. There were no obvious surgical contraindications and the patient received surgical treatment. However, the patient died of cardiac and respiratory arrest the next day after surgery. It was believed that the possibility of pulmonary embolism was extremely small if the patient did not stay in bed for a long time and did not use anticoagulant drugs after the operation. The patient’s postoperative blood pressure increased, and the diastolic blood pressure was 146 ~ 160 mmHg. The preoperative cardiac color Doppler ultrasound showed changes in the heart structure. Before the patient died, the patient’s condition changed suddenly and developed rapidly. Therefore, we thought the most likely direct cause of the patient’s death was: cardiac respiratory arrest caused by cardiogenic disease. At present, there are few studies on the perioperative death of patients caused by hypertension. The reason may be that hypertension leads to changes in cardiac structure and function, which makes patients prone to sudden cardiac death after surgery. Moreover, hypertension may also increase the risk of arterial rupture and bleeding in patients with neck infections and tumors invading the carotid artery. Peter G Noordzij et.al found that increased preoperative glucose levels were associated with perioperative mortality in patients undergoing noncardiac, nonvascular surgery [24]. Perioperative myocardial infarction (MI) is the most frequent fatal cardiovascular complication during noncardiac surgery [24–26], however, the pathophysiology underlying perioperative MI is not completely clear. It can be explained by a number of possible reasons. First, as a result of reduced insulin tolerance, subsequent hyperglycemia, prediabetes patients may be at risk for cardiovascular disease [27]. Secondly, compared with normoglycemic patients, patients with impaired glucose regulation are more frequently diagnosed with hypertension, obesity or dyslipidemia. This cluster of risk factors, also known as the metabolic syndrome, is associated with adverse cardiovascular outcome [24, 27].

Through this study, we found that active airway management can reduce the risk of perioperative death in HNC, which may be related to the special anatomical structure of the oral and maxillofacial region. Oral and maxillofacial tumors can easily cause airway obstruction after surgery, which can lead to death from suffocation. Especially when the tumor is located at the base of the tongue, floor of the mouth, or next to the pharynx, the risk of airway obstruction increases due to some issues such as flap repair and tissue edema. When the patient had airway obstruction after operation, emergency tracheotomy was often unable to open the patient’s airway in time due to postoperative tracheal displacement, bleeding and unclear operation area. Therefore, it is safer to perform tracheotomy immediately after the radical resection of HNC, so that the patient can maintain a smooth airway and avoid emergency tracheotomy after the operation.

For patients with HNC, hypertension, hyperlipidemia, chronic obstructive pulmonary disease (COPD) and diabetes are the most common comorbidities when diagnosed, and the prevalence is increasing [28]. Diabetes and hypertension are considered to be significantly related to the prognosis of a variety of tumor diseases [29], which significantly reduces the five-year survival rate of colon cancer patients [29–31]. There were also literatures reporting that diabetes was associated with longer survival rates in patients with malignant tumors [32]. So far, the relationship between diabetes [33], hypertension, preoperative chemotherapy [34, 35] or postoperative wound infection [34] and the long-term survival rate of HNC is still inconclusive. Through further survival analysis, we found that postoperative wound infection, hypertension and advanced clinical stages were significantly related to the poor prognosis of HNC patients. In summary, hypertension, diabetes, prior radiotherapy, preoperative chemotherapy, advanced clinical stages and wound infection are risk factors for perioperative death in HNC patients, while airway management can effectively reduce the mortality. Hypertension and wound infection are significantly related to poor prognosis, which suggests that we can perform tracheotomy while completely resecting the lesion, avoid blood vessels damaged by cervical radiotherapy, effectively control blood pressure and blood sugar during the perioperative period, and actively carry out postoperative anti-infection treatment. All of these measures can effectively prevent perioperative death and increase the five-year survival rate. In addition, this study indicated that age is not a risk factor for perioperative death and it may be related to the development of anesthesia and nursing technology in recent years. Therefore, when elderly patients do not have multiple complications, doctors can expand the scope of surgery and completely remove the lesions instead of choosing palliative treatments.

## Conclusions

In conclusion, prior radiotherapy, previous chemotherapy, postoperative infection, advanced clinical stages, hypertension and diabetes are risk factors for perioperative mortality of head and neck cancer. And hypertension, postoperative infection and advanced clinical stages are significantly related to poor OS in HNC patients. The causes of death included acute heart failure, rupture of large blood vessels in the neck, hypoxic ischemic encephalopathy due to asphyxia, respiratory failure and cardiopulmonary arrest.

Our study has several limitations that are similar to some case–control studies relying on retrospective data collection. First, information on patient characteristics might have been missed because of observer bias prejudice. Secondly, multivariable adjustment for potential confounders is obviously limited to the available data elements. Unknown, unmeasured confounders might still be present. Thirdly, the sample size of the patients in our study is small. Therefore, these conclusions need to be further verified by multi-center, large-sample prospective experiments.

## Data Availability

All the data in the manuscript are available from the corresponding author on reasonable request.

## Abbreviations

HNC: Head and Neck cancer
POMR: Perioperative mortality rate
MI: Myocardial infarction
COPD: Chronic obstructive pulmonary disease
